# A Huge Adenomatoid Odontogenic Tumor of Maxilla

**DOI:** 10.1155/2012/317341

**Published:** 2012-03-13

**Authors:** Balasundari Shreedhar, Iqbal Ali, Anshita Agarwal, Sarwar Alam

**Affiliations:** ^1^Department of Oral Pathology, Career Post Graduate Institute of Dental Sciences & Hospital, Lucknow 226020, India; ^2^Department of Oral and Maxillofacial Surgery, Career Post Graduate Institute of Dental Sciences & Hospital, Lucknow 226020, India; ^3^Department of Oral Pathology and Microbiology, Career Post Graduate Institute of Dental Sciences & Hospital, Lucknow 226020, India

## Abstract

The adenomatoid odontogenic tumor (AOT) is a benign, nonneoplastic (hamartomatous) lesion with a slow progressing growth. It occurs in both intraosseous and peripheral forms. This paper reports the case of a female aged 16 years who presented with a swelling in anterior maxilla; canine was missing, and a supernumerary tooth was present in the mid line. Radiology revealed a well-defined radiolucent area associated with impacted canine and root resorption of adjacent teeth, which was diagnosed histopathologically as AOT. The patient was treated surgically and later rehabilitated with fixed prosthesis.

## 1. Introduction

According to the second edition of the WHO “*Histological typing of odontogenic tumors*,” adenomatoid odontogenic tumor (AOT) is defined as “A tumor of odontogenic epithelium with duct-like structures and with varying degrees of inductive change in the connective tissue. The tumor may be partly cystic, and in some cases the solid lesion may be present only as masses in the wall of a large cyst” [1]. AOT is classified under “odontogenic epithelium with mature, fibrous stroma without odontogenic ectomesenchyme” [2, 3].

## 2. Review of the Literature

AOT is a relatively uncommon distinct odontogenic neoplasm that was first described by Steensland in 1905 [4]. Dreibaldt in 1907 described it as “pseudoadenoameloblastinoma” [5]. Harbitz in 1915 reported it as cystic adamantoma [6] and Ghosh in 1934 described it as an adamantinoma of the maxilla [7]. Staphne in 1948 first recognized AOT as a distinct pathological entity [8]. Bernier and Tiecke were the first to publish a case using the name “adeno-ameloblastoma.” The superb photomicrographs in their later series of nine cases from the Armed Forces Institute of Pathology undoubtedly made a profound contribution to the eventual recognition that AOT is not merely a type of ameloblastoma [9]. In 1961, Gorlin et al. introduced the term “ameloblastic adenomatoid tumor.” Shafer et al. provided additional support for this. In 1968, Abrams et al. suggested the term “odontogenic adenomatoid tumor.” This paper was in press and was not available to Philipsen and Birn when they proposed the name “adenomatoid odontogenic tumor” in 1969 [10]. Shortly thereafter, the latter term was adopted in the initial edition of the World Health Organization (WHO) “*Histological typing of odontogenic tumors, jaw cysts and allied lesions*” in 1971 and was retained in the second edition in 1992.

Unal et al. in 1995 produced a list containing all nomenclatures for AOT reported in the literature [11] like adenoameloblastoma, ameloblastic adenomatoid tumor, adamantinoma, epithelioma adamantinum, or teratomatous odontoma. In 1999, Philipsen and Reichart presented a review based on reports published until 1997 which showed some interesting aspects regarding epidemiological figures of this tumor [12]. Most recently, Leon et al. [2, 13] described a multicentre study of both the clinicopathological and immunohistochemical features of 39 cases of AOT [13]. Subsequently, adenomatoid odontogenic tumor became the generally accepted nomenclature and apparently has facilitated effective management of patients who have this lesion ever since. The AOT accounts for 1–9% of all odontogenic tumors [14].

## 3. Case Report

**Figure 1:**
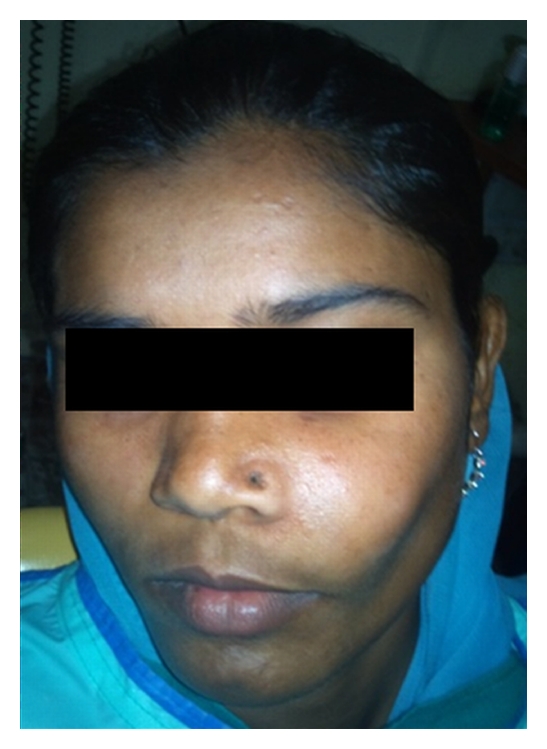
16-year-old female with a swelling on the left cheek.

 A 16-year-old girl presented with left anterior maxillary swelling of about 2 months duration. It was a slow growing swelling, without any other symptoms. On clinical examination, the left cheek was grossly deformed with normal overlying skin, and there was no neurological deficit over the affected area and no nasal discharge (Figure 1). The palatal vault was deformed, and egg shell crackling was felt. The oral mucosa over this area appeared healthy and asymptomatic, without evidence of infection. Canine was missing, and a supernumerary tooth was present in the midline. There was expansion of both buccal and palatal cortical plates of the left maxilla from the central incisor to the first molar on the same side.

**Figure 2:**
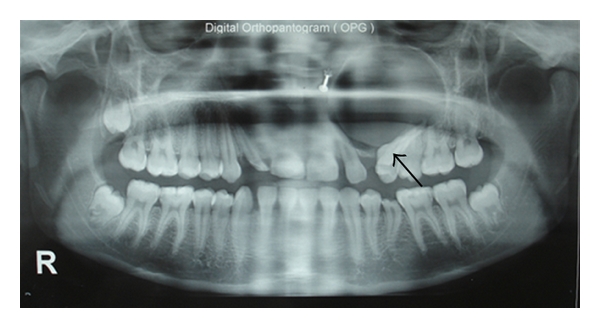
Oral pantomogram showing well-defined radiolucent cyst with canine tooth (arrow).

The radiographs showed a well-defined, unilocular radiolucency in maxilla with expansion and thinning of all its bony walls with the left upper canine tooth and without any evidence of calcifications (Figure 2). It also showed displacement of tooth and root resorption of first and second premolars. A clinical diagnosis of dentigerous cyst and adenomatoid odontogenic tumor was made. Other strictly radiolucent lesions worthy of consideration are keratocystic odontogenic tumor, ameloblastic fibroma, odontogenic myxoma, or central giant cell tumor as well as unicystic ameloblastoma as the age increases beyond 14 years [15].

**Figure 3:**
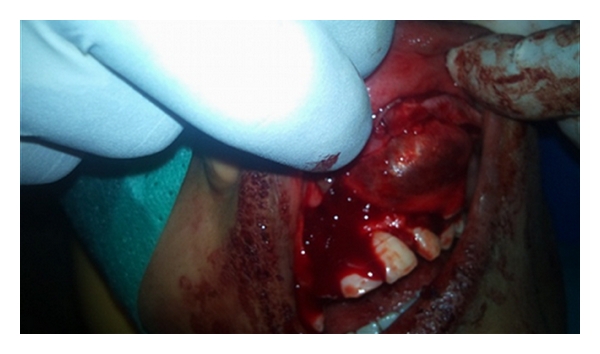
Intraoperative photograph showing the procedure undertaken for the removal of tumour.

The enucleation of the cyst was done under local anaesthesia along with the removal of the impacted canine, supernumerary, and first premolar tooth (Figure 3). It contained yellowish-brown-coloured fluid. After 4 weeks, root canal treatment was performed on the left second premolar and the first molar. A fixed prosthesis was given, and no recurrence was observed for the next 6 months.

**Figure 4:**
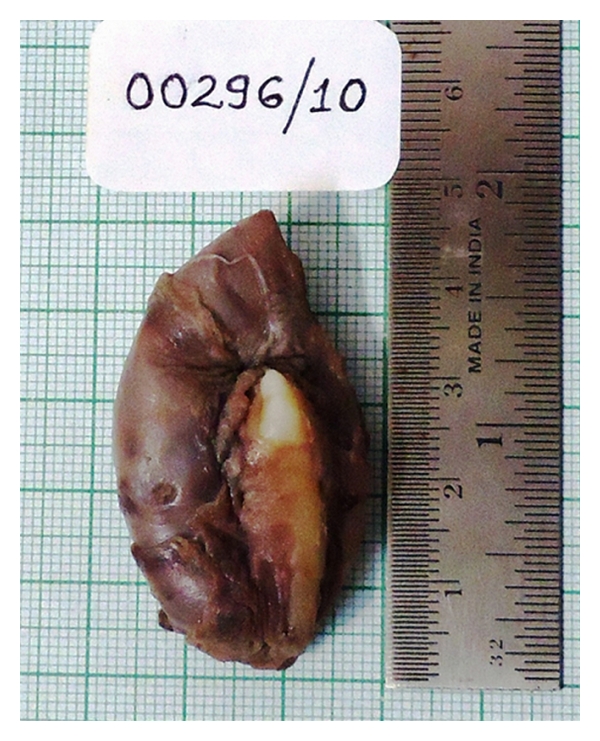
Gross examination revealed a cystic lesion showing the attachment of embedded canine tooth.

**Figure 5:**
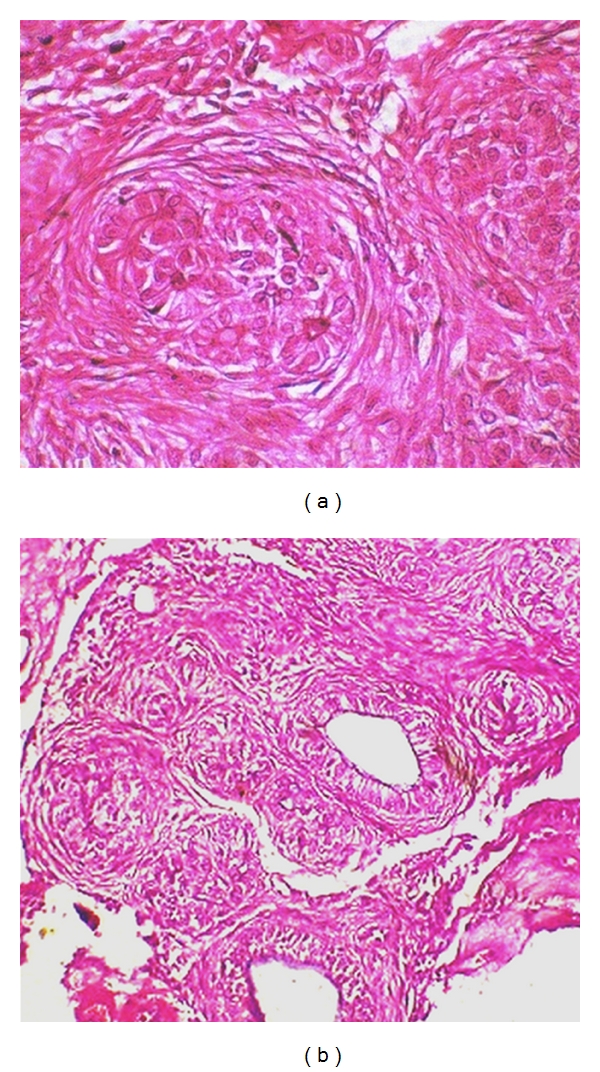
(a) Microscopic photograph showing tumor nodule composed of spindle-shaped or cuboidal epithelial cells forming rosette-like structures (H&E ×40). (b) Microscopic photograph showing duct-like structures of odontogenic epithelium lined by a single row of cuboidal or low columnar epithelial cells (H&E ×10).

Macroscopically, the specimen measured 4.5 × 3.5 × 4.0 cms with a smooth surface and was associated with a well-developed canine crown portion, circumscribed by cyst (Figure 4). The cystic region was brownish in color and contained a brownish fluid. Microscopically, The lesional tissue contained variable-sized solid nodules of columnar cells of odontogenic epithelium forming nests and rosette-like structures (Figure 5(a)). Between the epithelial cells and in the centre of the rosette-like configurations, eosinophilic amorphous material “tumour droplets” was present. The duct-like spaces were also seen and were lined by a single row of columnar epithelial cells, with the nuclei polarized away from luminal surface (Figure 5(b)). Connective tissue was fibrocellular with areas of hyalinization, moderate chronic inflammatory cell infiltrate, and marked vascularity. On these findings, histopathological diagnosis of AOT was made.

## 4. Discussion

The AOT is an uncommon cause of jaw swelling [15]. The tumor has three clinicopathologic variants, namely, intraosseous follicular, intraosseous extrafollicular, and peripheral. The extrafollicular type (24%) has no relation with an impacted tooth [16], whereas follicular type (73% of all AOT cases) is associated with an unerupted tooth as in the case we presented here, and the peripheral variant (3%) is attached to the gingival structures. Follicular and extrafollicular types are more common in the maxilla than in the mandible [12, 17], and most of the tumors involve anterior aspect of anterior maxilla [18, 19]. In our case, the tumor was a follicular intraosseous type and also found in the anterior region of the maxilla.

There is a slight female over male predilection, almost 2 : 1 [20]. If geographic/ethnic aspects are taken into consideration for the gender distribution, differences were observed between Asian and non-Asian races. Asian AOT cases (reported from Japan, India, China, Thailand, Taiwan, Sri Lanka, and Malaysia) show a female: male ratio of 2.3 : 1. If cases reported from Sri Lanka and Japan are considered separately, they show ratios of 3.2 : 1 and 3.0 : 1, respectively [18, 21], and this appears most often in the second decade of life [14, 22]. The sex and the age of the patient we described in this paper were consistent with the literature. The lesions are typically asymptomatic but may cause cortical expansion and displacement of the adjacent teeth [23], as in the case reported here. The origin of the AOT is controversial [24, 25]. Because of its predilection for tooth-bearing bone, it is thought to arise from odontogenic epithelium [9].

Although larger lesions have been reported in the literature [26, 27], the tumors are usually in the dimensions of 1.5 to 3 cms [28]. Radiographically, they usually appear unilocular and [26, 29] may contain fine calcifications [18], and irregular root resorption is rare [29]. This appearance must be differentiated from various types of disease, such as calcifying odontogenic tumor or cysts. The differential diagnosis can also be made with ameloblastoma, ameloblastic fibroma, and ameloblastic fibro-odontoma [15]. The patient we describe in this paper presented with size of AOT larger than 3.0 cms (4.5 cms) and also with root resorption with no evidence of calcification. Radiographically, it was not easily differentiated from dentigerous cyst, which usually occurs as a pericoronal radiolucency.

Histologically, the tumor is solid, and there is a cyst formation. The epithelium is in the form of whorled masses of spindle cells as well as sheets and plexiform strands. Rings of columnar cells give rise to duct-like appearance. Calcification is sometimes seen and may be extensive [14]. Interestingly, there are a few reports of AOT to occur with many types of cysts and neoplasms including dentigerous cyst [28], periapical cyst [16], calcifying odontogenic cyst, odontoma, ameloblastoma, and so forth [10, 27] and also about pigmented cells in AOT. However, all of these reported lesions did not show macroscopically visible pigmentation. Racial pigmentation probably plays an important role in such cases [30, 31].

The tumor is well encapsulated and showed an identical benign behaviour [12]. Therefore, conservative surgical enucleation produces excellent outcome without recurrence [32]. Our patient has been under followup for 6 months and was rehabilitated with fixed prosthesis.

## References

[1] Kramer IRH, Pindborg JJ, Shear M (1992). *WHO International Histological Classification of Tumors. Histological Typing of Odontogenic Tumors*.

[2] Barnes L, Eveson JW, Reichart P, Sidransky D (2005). *World Health Organization Classification, Tumours Pathology and Genetics, Head and Neck Tumours*.

[3] Jing W, Xuan M, Lin Y (2007). Odontogenic tumours: a retrospective study of 1642 cases in a Chinese population. *International Journal of Oral and Maxillofacial Surgery*.

[4] Steensland HS (1905). Epithelioma adamantinum. *Journal of Experimental Medicine*.

[5] Lucas RB (1984). *Pathology of Tumors of the Oral Tissues*.

[6] Harbitz F (1915). On cystic tumours of the maxilla, and especially on adamantine cystaadenomas (adamantomas). *Dental Cosmos*.

[7] Ghosh LS (1934). Adamantinoma of the upper jaw. *American Journal of Pathology*.

[8] Stafne EC (1948). Epithelial tumors associated with developmental cysts of the maxilla. *Oral Surgery, Oral Medicine, Oral Pathology*.

[9] Bravo M, White D, Miles L, Cotton R (2005). Adenomatoid odontogenic tumor mimicking a dentigerous cyst. *International Journal of Pediatric Otorhinolaryngology*.

[10] Rick GM (2004). Adenomatoid odontogenic tumor. *Oral and Maxillofacial Surgery Clinics of North America*.

[11] Unal T, Cetingul E, Gunbay T (1995). Peripheral adenomatoid odontogenic tumor: birth of a term. *Journal of Clinical Pediatric Dentistry*.

[12] Philipsen HP, Reichart PA (1999). Adenomatoid odontogenic tumour: facts and figures. *Oral Oncology*.

[13] Leon JE, Mata GM, Fregnani ER (2005). Clinicopathological and immunohistochemical study of 39 cases of adenomatoid odontogenic tumour: a multicentric study. *Oral Oncology*.

[14] Handschel JG, Depprich RA, Zimmermann AC, Braunstein S, Kübler NR (2005). Adenomatoid odontogenic tumor of the mandible: review of the literature and report of a rare case. *Head & Face Medicine*.

[15] Nigam S, Gupta SK, Chaturvedi KU (2005). Adenomatoid odontogenic tumor—a rare cause of jaw swelling. *Brazilian Dental Journal*.

[16] Philipsen HP, Srisuwan T, Reichart PA (2002). Adenomatoid odontogenic tumor mimicking a periapical (radicular) cyst: a case report. *Oral Surgery, Oral Medicine, Oral Pathology, Oral Radiology, and Endodontics*.

[17] Yilmaz N, Acikgoz A, Celebi N, Zengin AZ, Gunhan O (2009). Extrafollicular adenomatoid odontogenic tumor of the mandible: report of a case. *European Journal of Dental Education*.

[18] Toida M, Hyodo I, Okuda T, Tatematsu N (1990). Adenomatoid odontogenic tumor: report of two cases and survey of 126 cases in Japan. *Journal of Oral and Maxillofacial Surgery*.

[19] Swasdison S, Dhanuthai K, Jainkittivong A, Philipsen HP (2008). Adenomatoid odontogenic tumors: an analysis of 67 cases in a Thai population. *Oral Surgery, Oral Medicine, Oral Pathology, Oral Radiology and Endodontology*.

[20] Ajagbe HA, Daramola JO, Junaid TA, Ajagbe AO (1985). Adenomatoid odontogenic tumor in a black African population: report of thirteen cases. *Journal of Oral and Maxillofacial Surgery*.

[21] Mendis BRRN, MacDonald DG (1990). Adenomatoid odontogenic tumour: a survey of 21 cases from Sri Lanka. *International Journal of Oral and Maxillofacial Surgery*.

[22] Vera-Sempere FJ, Artes-Martínez MJ, Vera-Sirera B, Bonet-Marco J (2006). Follicular adenomatoid odontogenic tumor: immunohistochemical study. *Medicina Oral, Patología Oral y Cirugía Bucal*.

[23] Batra P, Prasad S, Parkash H (2005). Adenomatoid odontogenic tumour: review and case report. *Journal of the Canadian Dental Association*.

[24] Giansanti JS, Someren A, Waldron CA (1970). Odontogenic adenomatoid tumor (adenoameloblastoma). Survey of 111 cases. *Oral Surgery, Oral Medicine, Oral Pathology*.

[25] Tajima Y, Sakamoto E, Yamamoto Y (1992). Odontogenic cyst giving rise to an adenomatoid odontogenic tumor: report of a case with peculiar features. *Journal of Oral and Maxillofacial Surgery*.

[26] Larsson A, Swartz K, Heikinheimo K (2003). A case of multiple AOT-like jawbone lesions in a young patient—a new odontogenic entity?. *Journal of Oral Pathology and Medicine*.

[27] Khot K, Vibhakar PA (2011). Mural adenomatoid odontogenic tumor in the mandible—a rare case. *International Journal of Oral and Maxillofacial Pathology*.

[28] Sandhu SV, Narang RS, Jawanda M, Rai S (2010). Adenomatoid odontogenic tumor associated with dentigerous cyst of the maxillary antrum: a rare entity. *Journal of Oral and Maxillofacial Pathology*.

[29] Dayi E, Gürbüz G, Bilge OM, Ciftcioglu MA (1997). Adenomatoid odontogenic tumour (adenoameloblastoma). Case report and review of the literature. *Australian Dental Journal*.

[30] Takeda Y, Sato H, Satoh M, Nakamura S, Yamamoto H (2000). Pigmented ameloblastic fibrodentinoma: a novel melanin-pigmented intraosseous odontogenic lesion. *Virchows Archiv*.

[31] Buchner A, David R, Carpenter W, Leider A (1996). Pigmented lateral periodontal cyst and other pigmented odontogenic lesions. *Oral Diseases*.

[32] Motamedi MHK, Shafeie HA, Azizi T (2005). Salvage of an impacted canine associated with an adenomatoid odontogenic tumour: a case report. *British Dental Journal*.

